# 

**Published:** 1873-06

**Authors:** 


### Books and Pamphlets Received.

A Treatise on the Principles and Practice of Medicine. By Austin Flint, M. D., Professor of the Principles and Practice of Medicine and of Clinical Medicine in the Bellevue Hospital Medical College. Fourth edition; carefully revised. Philadelphia: Henry C. Lea. Buffalo: T. Butler & Son.

Text Book of Phhysiology, General, Special and Practical. By John Hughes Bennett, M. D., F. R. S. E., Professor of the Institutes of Medicine or Physiology, and Senior Professor of Clinical Medicine in the University of Edinburgh, etc. With twenty-one Photo-lithographic plates. Philadelphia: J. B. Lippincott & Co. Buffalo: H. H. Otis.

The Passions in their Relations to Health and Diseases. Translated from the French by Dr. X. Bourgeois, Laureate of the Academy of Medicine of Paris, etc. By Howard F. Dawson, A. M., M. D. Boston: James Campbell. 16mo., pp. 201.

Family Thermometry; a Manual of Thermometry for Mothers, Nurses, Hospitalers, etc. By Edward Seguin, M. D. New York: G. P. Putnam & Sons. 1873. 12mo. pp. 72.

The Function of the Eustachian Tube, in its Relation to the Renewal and Density of the Air in the Tympanic Cavity and to the Concavity of the Membrana Tympani. By Thomas F. Rumbold, M. D., St. Louis.

On Strictures of the Urethra. Results of Operation with the Dilating Urethrotome, with cases. By F. N. Otis, M. D., Clinical Professsor of Genito-urinary Diseases, College of Physicians and Surgeons, New York. Reprinted from the N. Y. Medical Journal, March, 1873.

Mayor's Message and Report of Board of Improvements of the City of Toledo, 1873.

THE BUFFALO

### Medical and Surgical Journal

PUBLISHED MONTHLY,

Containing Original Articles, Reports of Medical Societies and Hospitals, Editorial, Reviews, Correspondence, Army News, etc., etc.; including the usual variety of Medical Periodical Publications. Specimen copies sent on application.

Address Buffalo Medical & Surgical Journal, Buffalo, N. Y.

TERMS OF SUBSCRIPTION, - - $3.00 PER YEAR, IN ADVANCE.

